# A small, stainless-steel sieve optimized for laboratory beaker-based extraction of microplastics from environmental samples

**DOI:** 10.1016/j.mex.2019.07.012

**Published:** 2019-07-20

**Authors:** Ryota Nakajima, Dhugal J. Lindsay, Masashi Tsuchiya, Rie Matsui, Tomo Kitahashi, Katsunori Fujikura, Tomohiko Fukushima

**Affiliations:** Japan Agency for Marine-Earth Science and Technology (JAMSTEC), Japan

**Keywords:** Small stainless-steel sieve, Sample preparation, Time consuming, Underestimation, Non-plastic matter removal

## Abstract

Removing non-plastic materials is a mandatory process for studying microplastics in environmental samples, and non-plastic materials, both inorganic and organic matter, are often removed chemically through sequential processes. In the multiple chemical treatment processes, the samples need to be collected and the reagent removed at the end of each chemical treatment before the samples are again exposed to a different reagent in a separate container. This leads to a loss of microplastics to some extent. Here, we developed a new, yet simple, small sieve made of stainless-steel that can fit in a laboratory beaker (e.g. 200 ml volume), allowing it to be transferred as-is between chemical treatments of environmental samples, even being soakable in a beaker of acid solution. The collection rates of microplastics were significantly higher in the small stainless-steel sieve than the commonly used filter method for different size of microplastic particles. The use of the new sieve means the processes of rinsing off and filtering samples can be abbreviated throughout the entire process of non-plastic matter removal from environmental samples, contributing to a lower chance of microplastic loss. The time consumed in the sieve method was also significantly lower than for the filtering method due to the elimination of the collection and rinsing steps, thus the use of this sieve can reduce processing time for the samples. The new method is innovative in terms of reducing both the microplastic loss and processing time during chemical treatment processes.

• The method developed allows the lower chance of microplastic loss during chemical digestion process
• The method reduces the time of sequential processes during chemical digestion

## Graphical abstract

**Figure:**
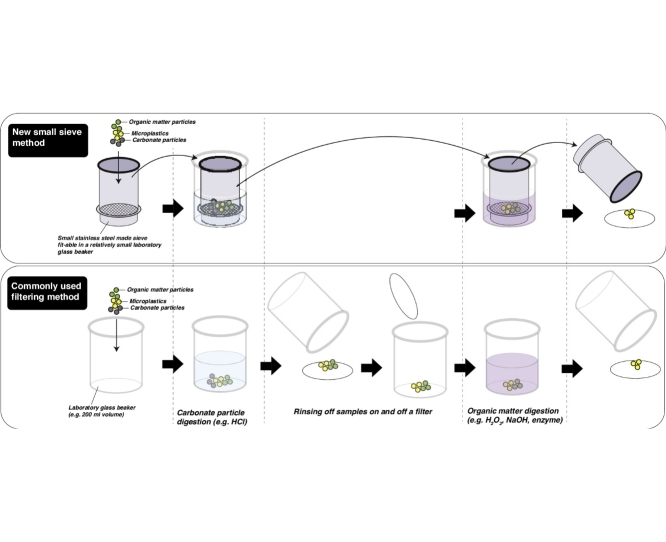

**Specifications Table**

**Table:** 

Subject Area:	Environmental Science
More specific subject area:	Microplastics
Method name:	Small stainless-steel sieve
Name and reference of original method:	No name is available to the original method
Resource availability:	Not available

## Method details

### Background

Removing non-plastic materials is a mandatory process for studying microplastics in environmental samples, because extraction and sorting of microplastics from raw environmental samples under the microscope is challenging due to the abundance of non-plastic materials [[1], [2], [3], [4]]. For example, surface samples collected by neuston or manta nets often contain large amounts of organic and inorganic particles, including detritus and zoo/phytoplankton, as well as sand [4,5]. This can interfere with the detection of microplastics under the microscope, and this is also true for sediment samples and animal gut samples, which can also contain a large amount of non-plastic materials [5]. Some of the inorganic materials such as sand can be separated by density separation using a heavy salt solution such as zinc chloride [6], while the other organic/inorganic particles are often removed (or digested) chemically, e.g., use of HCl for carbonate particles and H_2_O_2_, KOH, NaOH and enzyme for organic particles [[7], [8], [9]].

These chemical treatments for removing (or digestion) organic/inorganic particles usually take place in laboratory glassware, such as beakers or petri dishes, and are sequential processes [7]. At the end of each chemical treatment the samples need to be collected and the reagent removed before the samples are again exposed to a different reagent in a separate container [9]. The repeated procedures of the collection and rinsing off of samples on and off a filter or sieve invariably lead to a loss of microplastics to some extent. In this study, we report on our development of a small stainless steel sieve into which an environmental sample can be poured and then transferred in its entirety between different chemical reagents so as to prevent the loss of microplastics associated with sample transfer between different items of glassware. The use of this sieve also results in reduced processing time for the samples due to the elimination of the collection and rinsing steps.

### Materials and methods

**Fig. 1:**
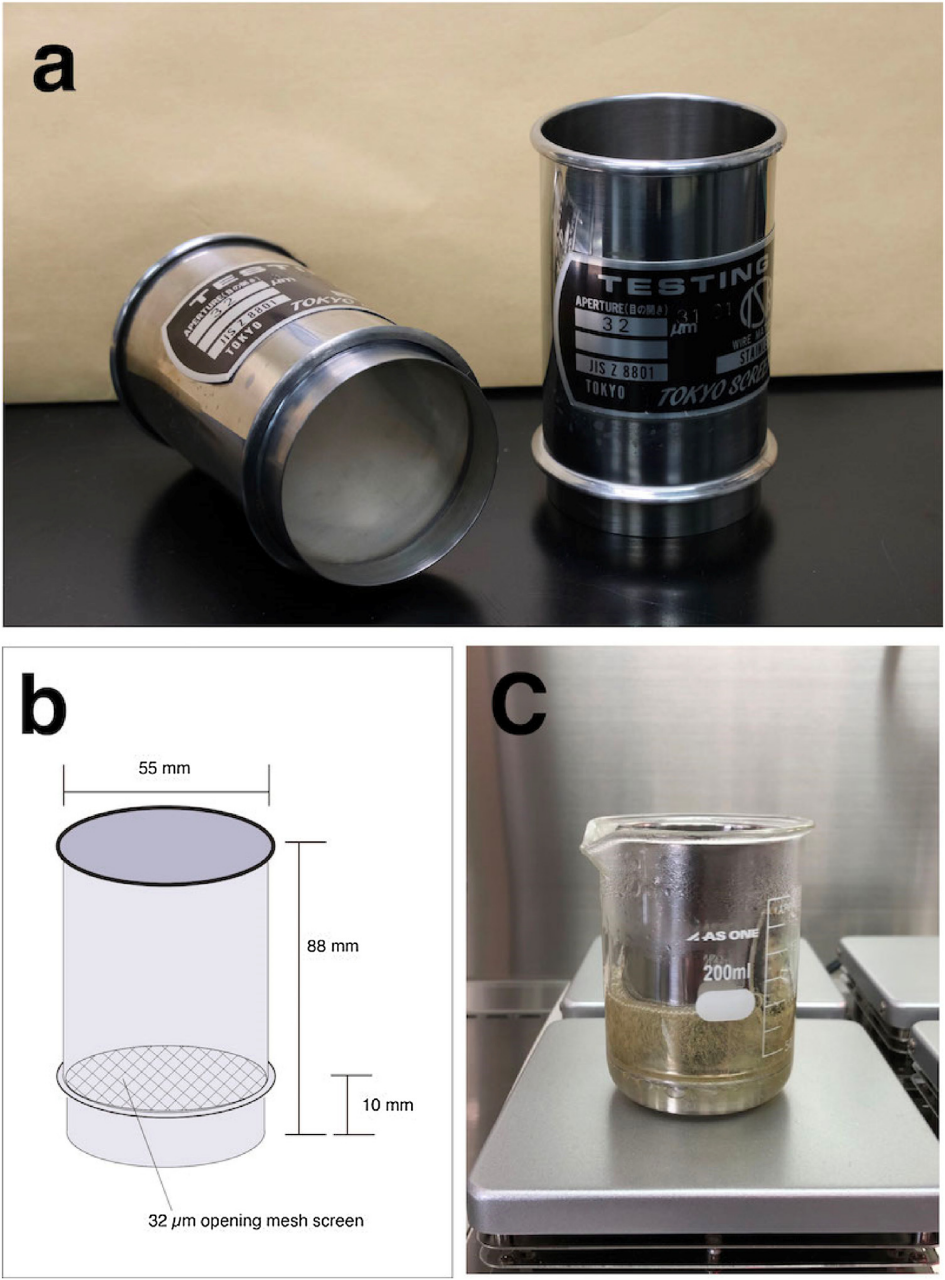
Newly developed small sieves optimized for laboratory beaker-based extraction of microplastics from environmental samples: (a) The small sieve is made of stainless steel, (b) diagram of the small sieve, (c) the small sieve is fit-able in a relatively small laboratory glass beaker (e.g. 200 ml volume), allowing it to be transferred as-is between chemical treatments of environmental samples, even being soakable in a beaker of acid solution.

We developed a small stainless-steel sieve that can fit in a relatively small laboratory glass beaker (e.g. 200 ml volume) (Fig. 1a). The sieve is a cylinder of 55 mm external diameter, is 88 mm high, and has a 32 μm opening mesh screen for filtering deployed 10 mm above the base of the cylinder (Fig. 1b). The entire sieve is made of stainless steel, making it effectively inert to chemical reactions, and allowing it to be transferred as-is between chemical treatments of environmental samples, even being soakable in a beaker of acid solution (Fig. 1c). A magnetic stirrer bar can be placed in the space below the mesh screen if desired.

In order to evaluate to what extent this small sieve can reduce the loss of microplastics, as well as how much it can reduce processing time, we compared the method using the new sieve to a previous method using filters for removing inorganic and organic particles. We prepared polystyrene microplastic particles of two size categories: 100–500 μm and 500–1000 μm. The microplastics were prepared by milling polystyrene plates with a plastic-grinder (PCL-2 M, Osaka Chemical) and then size-fractionated using stainless steel screens with a mesh opening of 100, 500 and 1000 μm. The microplastics (30 particles per replicate) were directly placed onto either the new sieve or on a filter (PTFE, Whatmann, 1.2 μm) with a glass filter holder. In total, 10 replicates of plastic samples were examined for each size class. We did not add plastic particles to other non-plastic materials, such as detritus, in order to directly compare the actual loss of microplastics between the sieve and the filter. Once placed either on the sieve or on the filter, the plastic samples were processed using exactly the same method as for environmental samples [7,8]. Firstly, the samples were processed for inorganic carbonate removal with 1 N HCl, before being soaked in 30% hydrogen peroxide with 0.5 M Fe(II) solution for organic matter removal [8]. The new sieve with microplastics was soaked in a beaker of 1 N HCl, while samples on filter were transported into the beaker containing 1 N HCl by rinsing off with the same HCl solution. After 20 min of reaction, the sieve was retrieved from the beaker of HCl and rinsed with a squirt bottle filled with MilliQ water, before being soaked in a beaker of Fe(II) + hydrogen peroxide solution. The other samples were filtered onto a new PTFE filter and rinsed with distilled water, and then all the residual solids were transferred into a beaker of Fe(II) + hydrogen peroxide solution by rinsing off with the same solution. The Fe(II) + hydrogen peroxide solutions were heated on a hot plate at 75 C degrees for 30 min [8]. After the organic matter removal process, the samples in the sieve, and the other samples in the beaker, were filtered onto another PTFE filter, and the collected microplastics were counted under a microscope. Artificially-incorporated microplastics were distinctive, both in color and shape, ensuring that only the experimentally-added plastics were counted.

The statistical difference in the collection rate of microplastics between the small sieve and the filter and the processing time between the small sieve and the filter were determined using Student’s t-test. Differences of P < 0.05 were considered significant.

### Method validation

#### The loss of microplastics retrieved

**Fig. 2:**
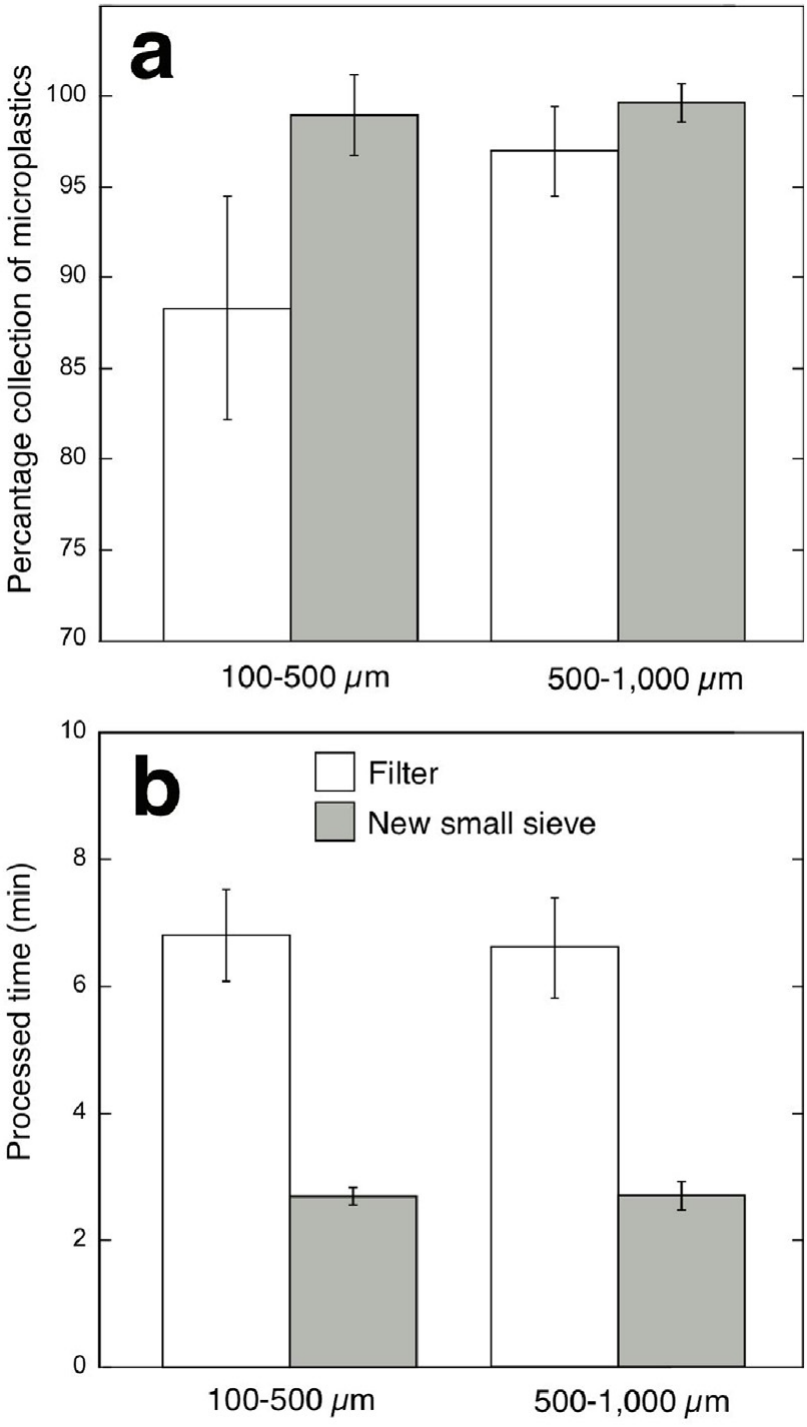
Comparisons of the new small sieve and classical filter methods in (a) microplastic collection rate and (b) processed time for different sized microplastics (100–500 μm and 500–1,000 μm).

The collection rates were significantly higher in the sieve than the filter method for both the smaller (100–500 μm) and larger (500–1000 μm) microplastics (p = 0.0055 for smaller and p = 0.0083 for larger microplastics) (Fig. 2a). The collection rate (%) in the small sieve was 99.0 ± 2.3% for smaller microplastics and 99.7 ± 1.1% for larger particles, while for the filter method it was 88.3 ± 6.1% and 97.0 ± 2.5%, respectively. The collection rates were lower for smaller particles in the filter methods (p < 0.0001), yet there was no significant difference between small and large particle retrieval in the sieve method (p = 0.41).

The repeated procedure of rinsing off samples retained on a filter, as well as filtering onto filters, may have increased the chance that microplastics were lost, probably due to splattering and/or adhesion of microplastics on the internal walls of the beaker or filter holder, and this may be more significant for smaller-sized microplastics. Studies employing this filtering method may therefore underestimate the number of microplastics. On the other hand, the small sieve method successfully reduced the loss of microplastics, thus contributing to more accurate counting of extracted microplastics.

#### Shortened processing time

The processing time throughout the inorganic and organic matter removal processes were compared between the small sieve and filtering methods (Fig. 2b). The time consumed in the sieve method was significantly lower (2.70 ± 0.13 min for small particles; 2.70 ± 0.22 for larger particles) than for the filtering method (6.80 ± 0.72 min for small particles; 6.60 ± 0.79 min for larger particles) (P < 0.0001 for both smaller and larger particles). In this study, without non-plastic materials, the time difference of about 4 min was simply due to the removal of the rinsing and filtering processes in the sieve method. This time difference should increase as the amount of non-plastic materials increases, as it requires more time to rinse them off and/or filter them.

To evaluate the efficiency of the new small sieve, we compared it with a classical filtering method in the present study. Some studies use a normal size stainless steel sieve such as used for meiobenthos study (e.g. 200–300 mm in diameter) [10], that does not fit in a glass beaker, for collection and rinsing off of samples throughout the inorganic/organic matter removal process [11]. The use of a normal size metal sieve may contribute to a shortening of the processing time compared to the classical filter method but there should be an associated loss of the retrieved microplastics due to the filtering process being of a similar nature. The normal size metal sieve is soakable in a relatively large bath of chemical solution, but it definitely requires a large amount of reactive acid solution and thus can be dangerous and costly. Thus, the use of a small sieve that can fit in a beaker has more benefit, since it both shortens the processing time and also reduces the chance of microplastics being lost, compared to the previous filtering method or the use of a normal-sized metal sieve. Although we tested the new sieve with artificially-incorporated microplastics the sieve can also be used for natural samples with microplastics. However, it should be pointed out that the new sieve had a 32 μm mesh screen, thus natural microplastics smaller than the mesh opening (e.g. 30 to several micrometers) would be lost by this method. The classical filtering method is probably better when the targeted microplastics are of a size smaller than 30 μm, though the mesh opening could even be smaller (e.g. 10 μm). It should also be noted that sometimes air can be trapped in the space below the mesh screen when putting the sieve in a beaker with solution, thus making a slit in the base of the sieve (below the mesh screen) would be helpful to release the air.

## Conclusion

We developed a new, yet simple, small sieve made of stainless steel that can fit in a laboratory beaker (e.g. 200 ml volume), and this allows chemical treatments of environmental samples containing microplastics in the sieve. The use of the new sieve means the processes of rinsing off and filtering samples can be abbreviated throughout the entire process of non-plastic matter removal from environmental samples, contributing to a lower chance of microplastic loss and a reduction in processing time. The new method is innovative in terms of reducing both the microplastic loss and processing time during chemical treatment processes.

## Supplementary material and/or Additional information

Not applicable.
